# Editorial: Accessible health programs promoting physical activity and fitness level

**DOI:** 10.3389/fpubh.2023.1163686

**Published:** 2023-03-08

**Authors:** Ziru Wang, Wendy Yajun Huang, Youcheng Liu, Guoxin Ni

**Affiliations:** ^1^School of Sport Medicine and Rehabilitation, Beijing Sport University, Beijing, China; ^2^Department of Sport, Physical Education and Health, Hong Kong Baptist University, Kowloon, Hong Kong SAR, China; ^3^Department of Family Medicine and Public Health Sciences, Wayne State University, Detroit, MI, United States; ^4^Department of Rehabilitation Medicine, The First Affiliated Hospital of Xiamen University, Xiamen, China

**Keywords:** physical activity, health program, fitness, implementation, influencing factors, accessibility, promotion

## Introduction

This Research Topic (RT) focused on different approaches to promote physical activity (PA), along with the underlying factors that may facilitate or hinder PA participation in diverse contexts and for different populations. The health benefits of PA have been supported by a significant number of studies (1, 2). However, most people in the world still do not meet the PA guidelines (3), and PA promotion is not routinely practiced in the clinical setting (4). An active lifestyle can be encouraged through a deeper understanding of its benefits and of how to effectively implement relevant strategies in usual clinical practice. PA promotion should be viewed as a collaborative effort that spans the person-environment system, while accessible facilities and services are one of the keys to building a more supportive environment, especially for people with disabilities, impairments, or low socioeconomic status. Targeting contributing factors and circumstances could improve public health communication for PA monitoring and promotion. To further develop interventions that target inclusive health programs and create a supportive environment, there is a need to focus on elements that are more practical to change as well as remove barriers to PA. Specifically, increasing the capacity of healthcare professionals and organizations can reinforce the importance of PA behavior in clinical practice pathways (5).

Collectively, this RT aims to provide state-of-the-art evidence on the approaches to the promotion of PA and fitness that can be incorporated into real-world practice. It contains 13 contributions on PA or fitness promotion, including original research (nine articles), reviews summarizing the up-to-date evidence (three articles), and a Delphi study developing evaluation indicators (one article).

## Factors influencing PA promotion for people with various socio-demographic characteristics

Health-related lifestyle changes, including PA, are impacted by society, especially during critical periods (e.g., the pandemic). A scarcity of accessible resources and support in society would impede PA participation and exacerbate the challenges faced by vulnerable individuals. In order to examine the consequences on PA changes of environmental factors such as limited access to exercise options and facilities, Zhou et al. compared the PA levels of middle-aged and older adults in China before and during the social distance restrictions imposed by the COVID-19 pandemic. Consistent with previous studies, their study indicated that groups with a lower level of socioeconomic status had a higher probability of PA reduction and that those with a higher level of income also found it hard to maintain positive PA habits during times of crisis. More importantly, their findings emphasized the importance of patient education for individuals with chronic diseases, as providing PA and exercise therapy prescriptions in the clinical setting has been shown to be beneficial in improving the health outcomes of these patients. This article highlighted the need to develop effective strategies (e.g., remote fitness/PA courses) that target these vulnerable populations, especially when routine PA activities are not accessible as usual.

Lábiscsák-Erdélyi et al. examined the impact of physical activity and its intensity on life satisfaction among Hungarian high school students, considering the role of gender and grade level. Results showed that physical inactivity had a negative effect on life satisfaction for both boys and girls, and was exacerbated by low perceived family wealth. Using vigorous PA intensity as the baseline, their results also suggested that vigorous PA is a simple and effective tool to reduce health inequalities among adolescents.

Liang et al. conducted a systematic review to investigate factors related to inclusive physical education (IPE) to expand PA promotion for students with special education needs (SEN) and in maintaining their health within school environments. The results of this review emphasized the need to target resource support and home-school collaboration, to fully utilize PA programs and facilities guided by trained educators. Their comprehensive summary aids in supporting educators and policymakers, which fills the gap between government policy and the current practice in China.

The development of technology and digitalization can be a two-edged sword. It boosts fitness trends, such as wearable technologies and artificial intelligence, but at the same time leads to an inactive and sedentary lifestyle. In the mini-review by Štajer et al., they attempted to provide a critical assessment of the present fitness trends and their shared effects on modern societies. Their results emphasized the demand for both advancements in knowledge and a more significant commitment of resources to increase PA globally.

To fully understand and increase participation in muscle-strengthening exercise (MSE) for all individuals, Gu et al. investigated potential correlates and the level of MSE for Chinese children and adolescents. They found that a range of demographic factors, including grade group, residence, ethnicity, and parental MSE, was associated with children's MSE. The results highlighted the significance of the development and implementation of school physical activity or sports policies.

## Evaluation and implementation of policy, strategy, and program

The floating population is always at a disadvantage to access social welfare and healthcare compared to residents. To more effectively promote equal access to basic public health services for migrants, the national essential public health services (NEPHS) in collaboration with relevant guidance have been developed and implemented; however, awareness and utilization remain low. A study conducted by Xu et al. found that the increased awareness of NEPHS had a beneficial effect on its utilization and the health of Chinese migrants. Their finding re-emphasized the important role of healthcare knowledge education to improve people's health literacy for building positive health-related behaviors, such as PA. Concerning the implementation of NEPHS, targeted services for various individuals along with health education activities are also recommended to maximize the benefits of such services.

Relevant PA policies in specific settings can positively impact PA behavior, particularly in the education system. To better adopt such policies, identifying barriers and facilitators of its implementation are the key steps. Using the Consolidated Framework for Implementation Research (CFIR), a cross-sectional study conducted by Wendt et al. provided insights about determinants of PA promotion policy adoption in the context of German elementary schools, including available resources and accessible knowledge and information.

To overcome the barriers to exercise, accessible community/home-based equipment and programs have constantly been developed. Hu et al. evaluated the generalizability and applicability of an exercise program (X-CircuiT) in terms of intensity and energy expenditure among sedentary people with various age groups. They provided a detailed recommendation of its use and confirmed its usability and positive effect on sedentary middle-aged and older adults.

Inactive adults in the workplace are another target population for the promotion of health and PA behavior. In Germany, companies have implemented workplace health promotion (WHP) to encourage PA. The study by Hoffmann and Schaller is the first to evaluate a communication strategy of PA measures in a cross-company network, using the mixed-methods analysis. They highlighted the importance of health communication strategies reaching the target group to raise people's awareness. The results offered insights into communication strategies to actively promote PA in the work setting, and have strong implications for the design of further strategies.

Sauter et al. investigated the specific effect (i.e., empowerment) of a long-standing health promotion approach in Germany targeting socially disadvantaged women. Their study confirmed the beneficial contribution of this PA project and suggests that establishing cooperative planning groups could be effective in involving socially disadvantaged women in the planning and implementation of PA programs. The findings suggest that focusing on socio-political environments at a local level to strengthen community capacities could sustainably implement programs that foster the health behaviors of minority groups.

## Training and evaluation of healthcare professionals

It is always required for healthcare professionals to implement high-level medical care for each patient. Therefore, continuous education is needed to upgrade their skills to better facilitate health services (e.g., PA prescription) implementation. Tung et al. conducted a systematic review of randomized controlled trials to explore the effectiveness of learning transfer in medical education and related training circumstances. Their findings suggested that continuous improvements for medical staff are still warranted to optimize their clinical decisions. Furthermore, a Delphi study conducted by Yi et al. developed evaluation indicators to better recruit and manage instructors to operate sports programs for people with disabilities. They highlighted the importance to evaluate professional sports instructors by incorporating Universal Design principles. Furthermore, public health crises, such as the COVID-19 pandemic, have presented challenges not only for patients but also for healthcare professionals. For example, Dai et al. conducted a longitudinal study to compare various elements of the education (i.e., infection containment control training) for dental residents during the pandemic, which provided meaningful implications for crisis-based training to facilitate their wellbeing.

## Conclusion

In conclusion, the studies included in this RT provide comprehensive insights into the various facets of promoting PA in a wide range of settings, catering to the needs and interests of different stakeholders. We hope this RT will inspire researchers to better design and implement new approaches targeting the highlighted contributing factors to build a more inclusive and accessible healthcare environment and to improve PA and fitness levels globally.

## Author contributions

All authors listed have made a substantial, direct, and intellectual contribution to the work and approved it for publication.
